# High intraspecific variability in the functional niche of a predator is associated with ontogenetic shift and individual specialization

**DOI:** 10.1002/ece3.1260

**Published:** 2014-12-02

**Authors:** Tian Zhao, Sébastien Villéger, Sovan Lek, Julien Cucherousset

**Affiliations:** 1 CNRS, Université Paul Sabatier, ENFA, UMR 5174 EDB (Laboratoire Évolution & Diversité Biologique)118 route de Narbonne, F-31062, Toulouse, France; 2 Université Toulouse 3 Paul Sabatier, CNRS, UMR5174 EDBF-31062, Toulouse, France; 3 Laboratoire Écologie des Systèmes Marins Côtiers UMR 5119 CNRS-UM2-IFREMER-IRD-UM1, Université Montpellier 2CC 093, 34 095, Montpellier Cedex 5, France

**Keywords:** Functional traits, niche, ontogeny, overlap, stable isotope analyses

## Abstract

Investigations on the functional niche of organisms have primarily focused on differences among species and tended to neglect the potential effects of intraspecific variability despite the fact that its potential ecological and evolutionary importance is now widely recognized. In this study, we measured the distribution of functional traits in an entire population of largemouth bass (*Micropterus salmoides*) to quantify the magnitude of intraspecific variability in functional traits and niche (size, position, and overlap) between age classes. Stable isotope analyses (*δ*^13^C and *δ*^15^N) were also used to determine the association between individual trophic ecology and intraspecific functional trait variability. We observed that functional traits were highly variable within the population (mean coefficient variation: 15.62% ± 1.78% SE) and predominantly different between age classes. In addition, functional and trophic niche overlap between age classes was extremely low. Differences in functional niche between age classes were associated with strong changes in trophic niche occurring during ontogeny while, within age classes, differences among individuals were likely driven by trophic specialization. Each age class filled only a small portion of the total functional niche of the population and age classes occupied distinct portions in the functional space, indicating the existence of ontogenetic specialists with different functional roles within the population. The high amplitude of intraspecific variability in functional traits and differences in functional niche position among individuals reported here supports the recent claims for an individual-based approach in functional ecology.

## Introduction

Biodiversity studies have primarily focused on the role of species richness (i.e., the number of species; Tilman 1997), although biodiversity has a multitude of facets (Gaston 1996; Purvis and Hector 2000) and the ecological characteristics and roles of species are not equal (Tilman 1997; Lavorel and Garnier 2002; Bolnick et al. 2011). During the last decade, there has been an increasing body of literature calling for the use of functional approach to understand and quantify biological diversity, notably in the general context of human-induced perturbations (Mouillot et al. 2013). Functional diversity approaches are based on the functional traits of species, that is, any biological attributes that impact fitness through effects on growth, reproduction, or survival of organisms (Violle et al. 2007), and it has been applied to address many ecological questions (Petchey and Gaston 2002; Mouillot et al. 2004; Mason et al. 2005). For animal species, these functional traits are typically obtained based on morphological measurements to estimate vital functions (e.g., locomotion and food acquisition in fish, Dumay et al. 2004; Mason et al. 2008; Villéger et al. 2010; Albouy et al. 2011 and foraging movements in birds, Ricklefs 2012).

In the meantime, population ecologists have reported the existence of intraspecific variability in morphological traits in animal taxa driven, for instance, by resource polymorphism, trophic specialization, ontogeny, or sexual dimorphism (Smith and Skulason 1996; Hjelm et al. 2000, 2001; Svanbäck and Eklöv 2002; Bolnick et al. 2003). However, despite the existence of such variations, functional ecologists have primarily focused on the differences in functional traits among species without accounting for the potential effects of intraspecific (i.e., between and within populations) variations in animal populations (Wilson et al. 1999; Ackerly and Cornwell 2007; Violle et al. 2012). This stands on the assumption that intraspecific variation was negligible compared to interspecific variation when studying functional ecology at the community level (McGill et al. 2006; Jung et al. 2010; Albert et al. 2011). Accordingly, low levels of intraspecific variation have been reported in the literature (Garnier et al. 2001; Dumay et al. 2004; Buckley et al. 2010). However, because intraspecific variations in functional traits can affect ecological interactions (Bolnick et al. 2011) and ecosystem functioning (Harmon et al. 2009; Rudolf and Rasmussen 2013a), it has been claimed that functional ecology should become more individual based than species based (McGill et al. 2006; Petchey and Gaston 2006; Cianciaruso et al. 2009; Violle et al. 2012).

This is especially true if intraspecific variation in functional traits is naturally present in wild populations. Indeed, within a population, the functional characteristics of individuals can change as individuals usually undergo morphological shifts during ontogeny (Miller and Rudolf 2011), and this intraspecific variations are ecologically relevant because they are often associated with ontogenetic shift in habitat and trophic niches (Ingram and Shurin 2009). Functional traits can also differ within life stages as individuals might exploit different ecological niches (e.g., resource polymorphism, Skulason and Smith 1995; Cucherousset et al. 2011). Depending upon their intensity, these two mechanisms can subsequently translate into different scenarios of niche overlap among life stages, ranging from no niche overlap between juveniles and adults to a high niche overlap whereby one life stage could be totally nested within the space of the other (Hammerschlag-Peyer et al. 2011). Previous studies have revealed that invertebrate consumers without metamorphosis could display an average of 40% niche overlap between life stages (Woodward and Hildrew 2002). It has suggested that this low ontogenetic niche overlap could decrease the stability of ecological networks (Rudolf and Lafferty 2011). However, there are still no empirical studies that have quantified the degree of variability and overlap in functional traits and the associated variation in trophic niche within a top predator species.

In this study, we quantified the distribution of functional traits describing food acquisition and locomotion (Villéger et al. 2010; Albouy et al. 2011) within a whole population of top predator fish to determine (1) the magnitude of intraspecific changes in functional traits during ontogeny; (2) functional niche position, size, and overlap between age classes; and (3) the association between functional and trophic (stable isotope) variability at the individual level.

## Materials and Methods

### Model species and sampling

Largemouth bass (*Micropterus salmoides*), a top predator freshwater fish species that has been introduced in more than 50 countries and reported to display ecological impacts (Cucherousset and Olden 2011), was selected as a model species. Largemouth bass undergo strong trophic niche shift during ontogeny as the species diet change from zooplankton to macroinvertebrates and prey fish (Post 2003). The sampled population was located in southwest France in a 1500 m^2^ private pond where angling was prohibited. This pond has been stocked approximately 15 years before sampling with largemouth bass and roach (*Rutilus rutilus*), a native cyprinid prey fish. Seine netting was used in July 2010 to collect largemouth bass and the pond was fully drained in October 2010 for maintenance purposes, ensuring that all individuals of the population were captured (*n*_total_ = 105). Collected specimens were euthanized using an overdose of anesthetic and preserved at −18°C.

### Data acquisition

In the laboratory, a set of 19 measurements describing the morphological characteristics of individuals was performed on each specimen directly using a scale and a digital caliper or through picture analyses (ImageJ). This set included mass (*M*), standard body length (Bl), body depth (Bd), caudal peduncle minimal depth (CPd), maximal caudal fin depth (CFd), caudal fin surface (CFs), eye diameter (Ed), distance between the center of the eye to the bottom of the head (Eh), total gut length (Gl), maximal gill raker length (GRl), head depth along the vertical axis of the eye (Hd), distance from the top of the mouth to the bottom of the head along the head depth axis (Mo), distance between the insertion of the pectoral fin to the bottom of the body (PFi), body depth at the level of the pectoral fin insertion (PFb), pectoral fin length (PFl), pectoral fin surface (PFs), body width (Bw), mouth depth (Md), and mouth width (Mw; Villéger et al. 2010; Albouy et al. 2011). Scales were collected in the antero-medial region of each individual for age determination (Britton et al. 2010), and individuals were subsequently grouped into three age classes: age-0, age-1, and ≥age-2. Finally, a sample of white dorsal muscle was collected on each specimen, oven dried (60°C for 48 h) and analyzed for stable isotope values (*δ*^13^C and *δ*^15^N) at the Cornell Isotope Laboratory (COIL, Ithaca, NY).

### Statistical analyses

We selected 16 complementary functional traits (Table 1) to reflect ecological functions of interest (i.e., multifaceted strategies associated locomotion and food acquisition) and which can be easily quantified on a large number of individuals (Dumay et al. 2004). Following these criteria and on the basis of published literature (Sibbing and Nagelkerke 2000; Mouillot et al. 2007; Schleuter et al. 2012; Reecht et al. 2013), functional traits were quantified using the aforementioned measurements. Functional traits described food acquisition (i.e., oral gape surface, oral gape shape, oral gape position, eye diameter, gill raker length, gut length), locomotion (i.e., eye position, body section shape, body section area, pectoral fin position, pectoral fin shape, caudal peduncle throttling, caudal fin shape, fins area ratio, fins area) or both (mass) in fish (Villéger et al. 2010; Albouy et al. 2011; Mouillot et al. 2013; details in Table 1). For instance, oral gape shape is associated to prey shape and capture. Specifically, individuals with lower oral gape shape tend to feed on benthic prey while individuals with higher oral gape shape tend to filter water for feeding (Karpouzi and Stergiou 2003). Pectoral fin position represents fish maneuverability and its position in the water column (Bellwood and Wainwright 2001; Bellwood et al. 2002; Wainwright et al. 2002; see details in Table 1 for other functional traits). Except mass, these functional traits are unitless ratio that are a priori independent of individual body size (Winemiller 1991; Villéger et al. 2010) to ensure that changes measured across age classes were not solely driven by changes in individual size.

**Table 1:** List of the 16 functional traits associated with food acquisition and locomotion (adapted from Villéger et al. 2010). The letter in brackets indicates the function associated with each trait (F, food acquisition and L, locomotion). Coefficients of variation (CV) measured in the population

Functional traits	Measure	Ecological meaning	CV, %
Mass (F/L)	log (*M* + 1)	Volume, muscle mass	24.71
Oral gape surface (F)	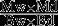	Maximum prey size or ability to filter water	18.61
Oral gape shape (F)	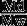	Prey shape and food acquisition	6.50
Oral gape position (F)	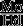	Position of prey in the water	16.03
Eye diameter (F)	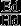	Prey detection	14.80
Gill raker length (F)	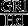	Filtration capacity or gill protection	31.39
Gut length (F)	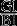	Digestibility of food	10.16
Eye position (L)	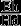	Position in the water column	11.51
Body section shape (L)	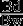	Position in the water column and hydrodynamism	5.03
Body section area (L)	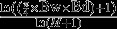	Mass distribution along the body and hydrodynamism	22.05
Pectoral fin position (L)	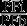	Maneuverability and position in the water column	6.73
Pectoral fin shape (L)	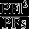	Propulsion and/or maneuverability	19.64
Caudal peduncle throttling (L)	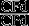	Swimming endurance	12.77
Caudal fin shape (L)	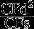	Endurance, acceleration, and/or maneuverability	18.14
Fins area ratio (L)	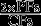	Swimming type (pectoral or caudal fin propulsion)	19.63
Fins area (L)	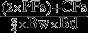	Endurance, acceleration, and/or maneuverability	12.23

*M*, mass; Bl, standard body length; Bd, body depth; CPd, caudal peduncle minimal depth; CFd, maximal caudal fin depth; CFs, caudal fin surface; Ed, eye diameter; Eh, distance between the centre of the eye to the bottom of the head; Gl, total gut length; GRl, maximal gill raker length; Hd, head depth along the vertical axis of the eye; Mo, distance from the top of the mouth to the bottom of the head along the head depth axis; PFi, distance between the insertion of the pectoral fin to the bottom of the body; PFb, body depth at the level of the pectoral fin insertion; PFl, pectoral fin length; PFs, pectoral fin surface; Bw, body width; Md, mouth depth; Mw: mouth width.

Intraspecific differences in functional traits were quantified using a multiple-trait approach. A synthetic multidimensional functional space was built by computing a principal components analysis (PCA) on the functional traits measured on all individuals (after scaling each trait to a mean of 0 and a standard deviation of 1; Villéger et al. 2008). The four-first principal components (eigenvalues > 1) were then used as synthetic axes (Villéger et al. 2008). Differences in niche position between age classes were tested using PERMANOVA on the first four axes. To quantify the effect of ontogeny on niche size, we calculated the functional niche size of each age class as the amount of space filled in the multiple dimensional functional space from the PCA axes (hull area, Villéger et al. 2008). Then the levels of functional niche overlap between three age classes were calculated following Villéger et al. (2013). As the number of individuals varied between age classes, we also computed bootstrapped functional niche size and overlap values (*n* = 10,000) based on the minimum number of individuals within the three age classes. In addition, we tested whether intraspecific variability affects the estimates of niche size obtained with a restricted number of individuals. We thus computed functional niche size on 10,000 random subsets of 15 individuals from the entire population. This sample size is similar to the number of individuals considered per species in study on functional diversity in fish communities (e.g., Mason et al. 2008; Villéger et al. 2010; Albouy et al. 2011; Mouchet et al. 2012). These bootstrapped functional niche size values were subsequently compared to the observed functional niche size of the entire population.

We also tested whether stable isotope values (*δ*^13^C and *δ*^15^N) and trophic niche position differ between age classes using PERMANOVA and Kruskal–Wallis tests. Stable isotope niche size and niche overlap were quantified using the convex hull area (TA) in *δ*^13^C-*δ*^15^N bi-plot space (Layman et al. 2007) for each age class. Although convex hull area could be affected by the number of individuals analyzed, it represents in this study the entire trophic niche in the population as all individuals were sampled. In addition, the core of the stable isotope niche was also quantified using standard ellipse area corrected for small sample sizes (SEA_c_; Jackson et al. 2011, 2012). Comparisons of stable isotope niche size between age classes were performed using Bayesian estimates of standard ellipse areas (SEA_B_; Jackson et al. 2011). Finally, correlations between each of the four PCA axes and each stable isotope value (*δ*^13^C and *δ*^15^N) were tested using Pearson correlations with Bonferroni corrections for multiple tests. All statistical analyses were conducted in R (R Development Core Team 2011).

## Results

Total length ranged from 62 to 139 mm, from 150 to 211 mm, and from 236 to 323 mm for age-0 (*n* = 33), age-1 (*n* = 64) and ≥age-2 (*n* = 8), respectively, and total length did not overlap between age classes. High intraspecific variations were observed for each of the 16 functional traits (mean coefficient variation: 15.62% ± 1.78% SE; Table 1). The four-first PCA axes explained 70.6% of the total inertia (PC1 = 28.9%, PC2 = 24.1%, PC3 = 10.3%, PC4 = 7.3%, respectively; Table 2). Specifically, PC1 was mainly driven by mass and functional traits related to locomotion; as PC1 values increased, individuals were more elongated and maneuverable (rounded pectoral fin shape) with a higher endurance (thicker caudal peduncles). PC2 was principally associated with functional traits related to food acquisition; as PC2 values increased, individuals displayed mouth in a more ventral position and laterally flattened with larger eyes and closer to the mouth.

**Table 2:** Pearson correlation coefficients between the four principal components analysis axes and the 16 functional traits. Significant *P*-values are in bold

Functional traits	PC1 (28.9%)	PC2 (24.1%)	PC3 (10.3%)	PC4 (7.3%)
Mass	**0.86**	**0.32**	−0.12	0.04
Oral gape surface	**0.30**	**−0.61**	0.08	**−0.21**
Oral gape shape	0.06	**0.20**	**−0.60**	−0.19
Oral gape position	**0.35**	**−0.74**	**0.20**	0.10
Eye diameter	**−0.59**	**0.65**	−0.05	−0.17
Gill raker length	0.08	**0.73**	**−0.34**	−0.04
Gut length	**0.38**	**0.53**	0.09	−0.19
Eye position	**0.41**	**−0.74**	0.15	0.02
Body section shape	0.12	0.08	0.11	**−0.85**
Body section area	**−0.79**	**−0.45**	0.16	−0.07
Pectoral fin position	−0.10	**−0.51**	0.01	**−0.52**
Pectoral fin shape	**−0.79**	0.18	**0.37**	0.04
Caudal peduncle throttling	**0.65**	**0.44**	**0.53**	0.00
Caudal fin shape	**0.66**	**0.41**	**0.50**	0.04
Fins area ratio	**0.52**	**−0.41**	**−0.62**	0.08
Fins area	**0.78**	−0.04	−0.01	−0.09

**Figure 1:**
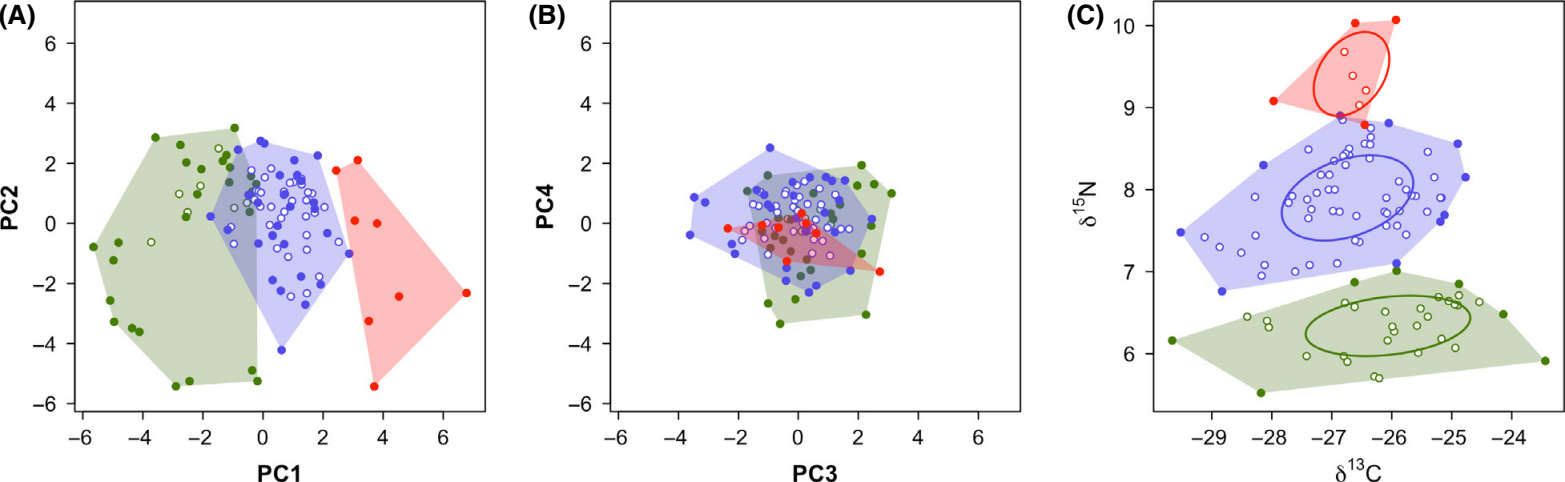
Distribution of the three age classes (green: age-0, blue: age-1, red: ≥age-2) in the functional and trophic spaces. (A) PC1 and PC2 of the functional space, (B) PC3 and PC4 of the functional space, and (C) stable isotope values (*δ*^13^C and *δ*^15^N). Colored polygons represent the niche size (convex hull area) of each age class, and filled points are vertices of the convex hull computed in four dimensions. Colored ellipses represent the standard ellipse area (SEAc) calculated for each age class based on stable isotope values.

The position of individuals in the functional space differed significantly among the three age classes (PERMANOVA *P* < 0.001, Fig. 1A and B). Observed functional niche size (hull area) decreased with age classes and was relatively low compared to the niche size of the entire population (Fig. 1A and B). While there were more age-1 (*n* = 64) than age-0 (*n* = 33) individuals in the population, they had a smaller observed functional niche size than age-0 individuals. The smallest observed functional niche size was displayed by ≥age-2 individuals (Table 3 and Fig. 1). Bootstrap tests revealed that, when considering only eight individuals, the functional niche size of age-0 and age-1 were not significantly different from the functional niche size of adults (Table 3). However, when considering 33 individuals, the functional niche size of age-1 was significantly lower than that of age-0 (Table 3). There was no functional niche overlap between ≥age-2 and age-0 classes and between ≥age-2 and age-1 classes. The functional niche overlap between age-0 and age-1 classes was 0.52%. Bootstrap tests considering only eight individuals in each age class also revealed a very low overlap between age-0 and age-1 classes (mean = 0.003% ± 0.0008% SE). When considering a random subsample of 15 individuals (i.e., 14.3% of the entire population), functional niche size estimate corresponded on average to only 7.9% (SE: ±2.5%) of the total functional niche size. This indicated that accounting for a restricted number of individuals in such a heterogeneous population disproportionately affects estimates of functional diversity in the population.

**Table 3:** Number of individuals in each age class, observed and bootstrapped functional niche size values considering only eight or 33 individuals (95% confidence interval) and trophic niche size (convex hull: TA; standard ellipse area: SEA_c_; Bayesian estimates of the standard ellipse area: SEA_B_) of the three age classes (age-0, age-1, ≥age-2)

		Functional niche			Trophic niche		
			
	*n*	Observed	Bootstrapped_n = 8_	Bootstrapped_n = 33_	TA	SEA_c_	SEA_B_
Age-0	33	243.69	1.45–47.81	–	5.41	1.53	1.82
Age-1	64	96.60	0.77–9.13	31.57–68.56	6.39	1.68	1.77
≥Age-2	8	5.94	–	–	1.34	0.95	1.69

Trophic niche position significantly differed between the three age classes (PERMANOVA, *P* < 0.001, Fig. 1C). Specifically, *δ*^15^N values (mean: 6.34 ‰ (±0.06 SE), 7.90 ‰ (±0.06 SE) and 9.41 ‰ (±0.17 SE) for age-0, age-1 and ≥age-2, respectively) significantly differed between age classes (Kruskal–Wallis test, *P* < 0.01, Fig. 1C), suggesting an increased trophic position during ontogeny. The origin of the carbon consumed by largemouth bass slightly but significantly changed during ontogeny as *δ*^13^C values (mean: −26.06 ‰ (±0.24 SE), −26.74 ‰ (±0.14 SE) and −26.67 ‰ (±0.21 SE) for age-0, age-1, and ≥age-2, respectively) differed significantly between age classes (Kruskal–Wallis test, *P =* 0.01, Fig. 1C). Interestingly, within each age class, the range of *δ*^13^C values was high, but the trophic niche size of each age classes was relatively low compared to the entire population. Age-0 individuals (TA = 5.41 and SEA_c_ = 1.53) filled slightly less trophic space than age-1 individuals (TA = 6.39 and SEA_c_ = 1.68) and these two age classes filled more trophic space than ≥age-2 (TA = 1.34 and SEA_c_ = 0.95; Table 3; Fig. 1C). Although these differences in trophic niche size were not significant between age classes (age-0: SEA_B_ = 1.82, age-1: SEA_B_ = 1.77, ≥age-2: SEA_B_ = 1.69, *P* > 0.05), each age class could also be considered to occupy distinct trophic niche as there was no or only little trophic niche overlap between age classes (age-0 vs. age-1: 0 and 0%; age-0 vs. ≥age-2: 0 and 0%; age-1 vs. ≥age-2: 0.28 and 0% for TA and SEA_c_, respectively).

**Figure 2:**
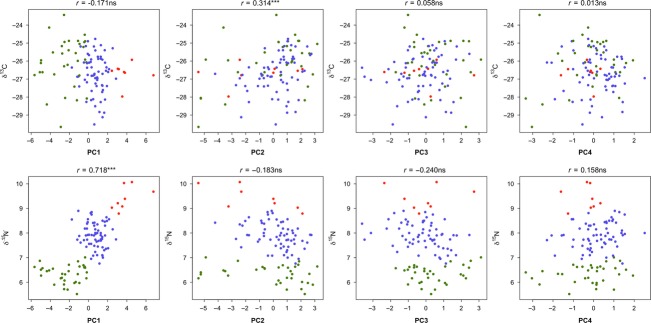
Pearson correlations between individual values on the four principal components analysis axes and stable isotope values (*δ*^13^C and *δ*^15^N, trophic niche; green = age-0; blue = age-1; red = ≥age-2). ns: not significant; ***: *P* < 0.006 (Bonferroni correction).

*δ*^13^C values were significantly and positively correlated with PC2 (Pearson correlation, Bonferroni correction, *r* = 0.31, *P* < 0.006, Fig. 2). *δ*^15^N values increased significantly with PC1 (Pearson correlation, Bonferroni correction, *r* = 0.72, *P* < 0.006; Fig. 2). PC3 and PC4 were not significantly correlated to *δ*^13^C (Pearson correlation, Bonferroni correction, *r* = 0.06, *P* = 0.560 and *r* = 0.01, *P* = 0.894, respectively) and to *δ*^15^N values (Pearson correlation, Bonferroni correction, *r* = −0.24, *P* = 0.014 and *r* = 0.16, *P* = 0.109, respectively).

## Discussion

The present study demonstrated that the variation in the functional attributes of individuals within a top predator population was high, principally driven by ontogenetic shifts (differences among age classes) coupled to interindividual variability (differences within age classes). Overall, the level of overlap in functional niche among the three age classes was null or extremely low. Specifically, the patterns of ontogenetic niche shift demonstrated that age-0 individuals overlapped only slightly with age-1 individuals that displayed a totally distinct niche than ≥age-2 individuals. Additionally, we found that age-0 and age-1 individuals significantly differed in terms of functional niche size, that is, the amount of space occupied in the functional space. All of these observations indicated that these three age classes should be considered as distinct functional entities when investigating the functional properties of populations or the functional diversity of communities as this source of variation could disproportionally affect the estimates of functional diversity. Furthermore, variations of 16 functional traits within the population were associated with significant changes in stable isotope values. A vast majority of animal species may modify their trophic resource use during ontogeny (Werner and Hall 1988) and this source of intraspecific variations can affect ecosystem functioning (Rudolf and Rasmussen 2013a). In the present study, despite the absence of differences in trophic niche size, there was no or extremely low overlap between the three age classes. Overall, these results demonstrated that largemouth bass should be considered as an ontogenetic ecological specialist, which could potentially reduce the level of stability in ecological networks such as food webs (Rudolf and Lafferty 2011).

Particularly, there was a strong association between ontogenetic trophic niche shift and several functional traits. The significant positive relationship between *δ*^15^N values and PC1 indicated that in addition to increased mass, most functional traits related to locomotion varied significantly. This might indicate change in locomotion attributes associated with foraging behavior and the mobility of prey encountered during ontogeny as largemouth bass diet shits from consuming zooplankton, macroinvertebrates to fish (Post 2003). During ontogeny, individuals displayed deeper body, thicker caudal peduncles, and more rounded pectoral fins and become more elongated and maneuverable with a higher endurance (Bellwood and Wainwright 2001; Wainwright et al. 2002) to mainly forage on prey fish in the pelagic area (Blake 2004), leading to higher *δ*^15^N values. Such morphological changes during ontogeny are relatively common in predatory fish (Amundsen et al. 2003; Johansson et al. 2006; Zimmerman et al. 2009). Interestingly, age-0 and age-1 individuals displayed a relatively wide range of *δ*^13^C values which indicated that, within the same age class and with similar *δ*^15^N values, individuals consumed prey with different origins such as aquatic and terrestrial invertebrates (Cucherousset et al. 2007). These results therefore suggest the potential existence of trophic specialization associated with differences in functional traits within life stages (Wilson et al. 1996; Svanbäck and Eklöv 2002). Moreover, the positive relationship between *δ*^13^C values and functional traits related to food acquisition suggested that individuals with higher PC2 values (i.e., larger eyes which were closer to the head, ventral position, and laterally flattened mouth) preying upon invertebrates or insects in the littoral zone (Winemiller 1991; Karpouzi and Stergiou 2003; Pouilly et al. 2003), leading to higher *δ*^13^C values.

Several studies have demonstrated that intraspecific variation in functional traits could be negligible compared to interspecific variation (McGill et al. 2006; Jung et al. 2010; Albert et al. 2011). At the opposite, the magnitude of intraspecific variation in functional traits observed in the present study suggests that, irrespective of its drivers, it should be considered (Albert et al. 2011, 2012; De Bello et al. 2011; Rudolf and Rasmussen 2013a). Therefore, we argue that intraspecific variability in predatory species should be explicitly accounted for when studying functional diversity of communities as distinct ecological entities can actually be discriminated within a population (Violle et al. 2012). Furthermore, as these ontogenetic differences can affect community structure and ecosystem functioning (Rudolf and Rasmussen 2013b), intraspecific variability in functional traits is likely to be important in ecosystem ecology. Using mean functional trait values across all age classes to estimate the diversity of communities is not appropriate as functional traits variation is dynamical and related to changes of population demographic structure (Rudolf and Rasmussen 2013b; Rudolf et al. 2014). All of these findings reinforce the need of quantifying intraspecific functional variability and the general idea that a shift from “species level” to “individual level” may enhance the ability of ecologists to understand and predict ecological patterns and processes (Bolnick et al. 2011; Violle et al. 2012).
